# Commencement Exercises of the Southern Medical College, Session 1883-1884

**Published:** 1884-03-20

**Authors:** 


### EDITORIALS AND MISCELLANEOUS.

### *COMMENCEMENT EXERCISES OF THE SOUTHERN MEDICAL COLLEGE, SESSION 1883–1884.*

The Commencement Exercises of the above new and rising Institution in Atlanta took place at the Opera House on the evening of the 25th of February. The audience was the largest and most intelligent ever, perhaps, assembled on a similar occasion in the city of Atlanta.

The graduating class was admirably arranged in two columns, fronting obliquely on either side, with the Faculty, the Board of Trustees and the Orator of the Occasion in the back centre. The stage was decorated in a beautiful and artistic style. A pyramid of evergreens and flowers was placed in the centre, with a beautiful floral inscription of the letters, S. M. C.

The programme was opened with an appropriate and eloquent prayer by the Rev. C. M. Beckwith, D.D., of the Episcopal Church.

The report of W. P. Nicolson, the Dean, was then read, as follows:

*To the President and Board of Trustees:*

I have to report at the close of the session of 1883–4 that there have been in attendance upon lectures and matriculates one hundred and two students, representing all sections. There has been every effort on the part of the Faculty to make the course more complete than ever before, and there has been a strong tendency towards more rigid examinations and higher requirements. There has been a reduction in the number of graduates, due to many causes—sickness, sudden demands at home, etc. The faculty have labored earnestly to discharge the obligations resting upon them in every way, and we are sure the class, as a whole, appreciate it. I am able to report a far better clinical course, due in a large measure to the facilities afforded by the Ivy Street Hospital.

We present to you to night for the degree of doctor of medicine the following gentlemen, who have, after a satisfactory examination, been found worthy of the degree of doctor of medicine.

The Dean, after reading his report, proceeded to hand Diplomas to the following graduates, to-wit:

Idus P. Allred,
Joseph L Baker,
Thomas H. Bonner,
James C. Borders,
Richard S. Bradley,
John F. Bruce,
Peter W. Butler,
James H. Duggan,
Jefferson D. Duke,
Thomas A. Fowler,
John B. Goodwin,
John T. Harris,
Andrew M. Jamison,
Isaac C. Johnson,
Charles W. Kirby,
Charles B. Lee,
William R. McCrary,
Charles E. McPhelemy,
Roger A. Mallory,
Augustus C. Murchison,
Francis J. Nelson,
J. W. C. Pettis,
Robert F. Pharr,
John W. Pinkston,
J. W. V. R. Plummer,
Thorp P. Roberts,
John C Smith,
John M. Suttles.

Prof. Thomas S. Powell, President of the Board of Trustees, then conferred the degrees in due form, closing with the following appropriate, beautiful and eloquent address to the graduates:

*Gentlemen:*

A peculiar interest, if not anxiety, is always awakened in the hearts of a young man's friends when they see him about to take the first steps in the untried path of a professional life.

But when we look upon a number, as on this occasion, with all preparations made, and their loins girt about with will and purpose to pursue the responsible and arduous career of a practitioner of medicine, that interest and anxious solicitude is increased tenfold.

There is also something pathetic to me in the beginning of a life-work by the young, and their eager ambition to make a noble struggle for success and secure a worthy position before the world.

I find myself asking if their path will be a rugged one of thankless toil, with scarcely a flower by the wayside to cheer and brighten with its beauty and fragrance, or will their feet more often tread pastures of verdure and bloom, where clear waters leap in ripples of glee and birds sing in the shade of green trees, refreshing the weary traveler into forgetfulness of past toil and giving him strength for the labor of the journey still beyond.

No wonder, my young professional brethren, that, with full hearts and enthusiastic emotions, your friends spontaneously cry out to you, "God speed you on your way."

God is always good, and He gives to youth sanguine hopes and joyful expectations, paints their skies the color of the rose, makes their fancy glow with golden dreams, and thus fortifies them with vigor for the toil that life may have in store for their inexperienced hands.

And I would not throw the shadow of a cloud over these beaming skies, nor chill, in the least degree, the enthusiastic ardor of youth's bright hopes. On the contrary, my young friends, I would say to you, always be enthusiastic in your work and in all noble purposes.

Without earnestness and consecrated efforts, you cannot succeed, for this Divine spark of enthusiasm is the secret of the eloquence of work as well as of words, and, with electric force, it scatters obstacles and opposition before it that would not be removed by any other agent.

Also, be ambitious. Your professional studies, instead of being ended, are but scarcely begun, and, while your ambition should be to do your humblest duty nobly, let me exhort you to emulate and strive for the very highest attainments in your work and your life.

But do not be so solicitous for reputation as for character.

When you feel sure you are right in your convictions, be firm in maintaining them against all opposition and aggression.

With solidity for the granite basis of your character, build upon it the noble principles, the high purposes, and all the fair, but enduring proportions of an exalted life.

Let me ask you to have a deep reverence for all sacred things, and to keep yourselves unspotted from the world; for, whatever may be said of its members, your profession, itself, stands a head and shoulders above all others of an earthly character, and each one of you will do your part in ennobling and exalting that profession or debasing it in the eyes of the world and the medical fraternity. I would have you to love, as well as honor, your profession; to be jealous of its position and its rights; but never let a spirit of avarice deter you from exercising your highest professional skill in behalf of the suffering poor as freely and courteously as toward your wealthiest patrons. This is, emphatically, the noblest prerogative of the physician.

Next to reverence for your Creator, ever hold sacred the name of woman in your hearts and upon your lips. Your profession must, necessarily, bring you in close contact with her mental and physical suffering. Be gentle, be kind to her, and give her your sympathy as well as your skill. Be to her a true friend and a wise counsellor, and let her see that with profound respect as well as admiration you regard the wonderful and beautiful combinations of her nature and the Divine appointment of her destiny in the world.

When your career is ended here, my young friends, may each and all of you have the blessed hope of a life to come in that bright world beyond the star-spangled skies.

The next step on the programme was the valedictory address by Dr. P. W. Butler in behalf of the class. The address was, in all respects, an able and admirable production, a considerable portion of it being in original verse. The whole was well and happily delivered. It evinced marked and unusual ability on the part of the speaker, and elicited the profound attention and the repeated applause of the audience.

*The Annual Address* was next delivered by the Rev. M. B. Wharton, D.D His subject, "The Relations between the Physician and the Minister," was happily chosen and, though extemporaneously delivered, was most ably and eloquently handled. He said that the offices of the minister and physician were combined in the olden time—the elders prayed and the physicians anointed. The minister and the physician are found bravely together amid devastating epidemics and scenes of death. The physician should be a man of integrity, virtue and morality. The mission ot the medical man, like that of the minister, was one of mercy and of love. Both ministers and physicians are alike execrated and contemned who refuse their offices to the poor.

There is no wrangling between physicians and ministers, and the speaker bestowed a high compliment upon the doctors for their kindness and liberality in not charging ministers.

His allusions to the Southern Medical as a Home Institution, and, therefore, to be supported, were well put and well sustained, and his mention of the American in Europe who could and would find nothing equal to his own native America furnished a humorous and happy illustration of the point taken, and brought down the House with repeated laughter and applause.

Many other important points were made, which our space will not permit us to mention. Suffice it to say that the address was, in all respects, among the best, most appropriate and most eloquent ever delivered on any like occasion in Atlanta.

After the above address the Dean, as representative of the Faculty, delivered the first and second honors for proficiency in all the branches: First honor to Dr. P. W. Butler, of Georgia. Second honor to I. C. Johnson, of Georgia.

Prizes in the special branches were then conferred upon the following graduates by the separate professors:

*In Eye and Ear*, to Dr. R. A. Mallory by Professor A. G. Hobbs.
*In Operative Surgery*, to Dr. P. W. Butler by Prof. G. G. Crawford.
*In Practice*, to Dr. I. C. Johnson by Professor W. D. Bizzell.
*Principles of Surgery*, to Dr. P. W. Butler by Prof. Jno. Thad Johnson.
*Materia Medica*, to Dr. J. H. Duggan by Professor G. G. Roy.
*Chemistry*, to Dr. P. W. Butler by Professor J. A. Burns.
*Physiology*, to Dr. Wm. R. McCrary by Professor R. C. Word.
*Obstetrics*, to Dr. J. B. Goodwin by Professor T. S. Powell.

The several parts of the Programme were interspersed with music, the most artistic and beautiful; floral gifts were profusely distributed by the ladies to the young graduates, and the entire occasion was the best gotten up, the most attractive and the most interesting of the kind we have ever witnessed in Atlanta.

